# Should thrombopoietin receptor agonists be used for chemotherapy-induced thrombocytopenia?

**DOI:** 10.1016/j.rpth.2025.102980

**Published:** 2025-07-22

**Authors:** Hanny Al-Samkari

**Affiliations:** 1 Division of Classical Hematology, Mass General Brigham Cancer Institute, Massachusetts General Hospital, Boston, Massachusetts, USA; 2 Harvard Medical School, Boston, Massachusetts, USA

**Keywords:** avatrombopag, bleeding, chemotherapy-induced thrombocytopenia, relative dose intensity, romiplostim, thrombopoietin receptor agonist

## Abstract

Chemotherapy-induced thrombocytopenia (CIT) is a common complication of cancer therapy for solid tumors that results in increased bleeding risk and chemotherapy dose reductions, treatment delays, and agent discontinuation. Unlike other chemotherapy-induced cytopenias, CIT remains without any licensed therapies in most of the world. Multiple thrombopoietin receptor agonists (TPO-RAs) have been approved for other thrombocytopenic indications, however, and are widely available, offering an accessible option for CIT management. In this Research and Practice in Thrombosis and Haemostasis Forum article, the historical reasons for the current state of CIT treatment are explained, the potential benefits and risks of TPO-RA use in CIT are discussed, and the patient populations who are likely to benefit and not benefit from TPO-RA support are described.

## 1 Introduction

Pharmacologic supportive care is a critical pillar of modern solid tumor oncologic treatment, allowing patients to better tolerate the side effects of chemotherapy, minimizing the risks of chemotherapy-related adverse events, and ultimately improving oncologic outcomes. Most pharmacologic supportive care in oncology is noncontroversial. Aggressive antiemetic prophylaxis and the use of powerful antiemetic medications for breakthrough nausea are one example. Likewise, primary and secondary prevention of febrile neutropenia with granulocyte-stimulating factors is ingrained into the standard of care, allowing for the safe maintenance of the relative dose intensity (RDI) of chemotherapy while minimizing the risk of hospital admission and death from febrile neutropenia. Prior to the advent of modern antiemesis treatment or granulocyte growth factors, RDI reduction in the form of chemotherapy dose reduction, chemotherapy treatment delays, and chemotherapy agent discontinuation for severe nausea or neutropenia and its complications was the accepted standard of care. However, in 2025, with agents widely available to treat and prevent these once treatment-limiting complications, opting to reduce the RDI for neutropenia or nausea—without even attempting the use of pharmacologic supportive medications to address the issue first—would be viewed as suboptimal care at best and medical malpractice at worst (particularly in the very common case of patients being treated for potentially curable cancer). However, when it comes to chemotherapy-induced thrombocytopenia (CIT), anywhere in the world outside of China, RDI reduction is commonly performed without hesitation to minimize the risk of profound thrombocytopenia and major bleeding, even in 2025, when multiple thrombopoietic agents are available. Why is this the case?

## 2 How Did We Get Here?

In the 1990s, hematopoietic growth factors emerged with the prospect of transforming the management of hematologic toxicities of chemotherapy. Filgrastim (granulocyte colony-stimulating factor [G-CSF], Neupogen; Amgen) was approved by the US Food and Drug Administration (FDA) in 1991 and revolutionized the management of chemotherapy-induced neutropenia. Epoetin alfa (Procrit; Amgen) was approved by the US FDA in 1993 for the management of chemotherapy-induced anemia. To complete the trifecta of growth factor approvals, oprelvekin (recombinant human interleukin-11, Neumega; Wyeth), a cytokine with thrombopoietic effects that did not agonize c-MPL, the thrombopoietin receptor, was approved in 1997 for the management of CIT in patients with solid tumors. G-CSFs were further refined and heavily integrated into the standard of care, where they remain a staple in oncologic supportive care for patients with solid tumors and hematologic malignancies. Erythropoiesis-stimulating agents were implicated in causing solid tumor progression and largely abandoned in oncology on the basis of trials with high hemoglobin targets and large erythropoiesis-stimulating agent doses that led to poor outcomes, despite the fact that more recent trials using more modest doses targeting typical chronic kidney disease hemoglobin levels did not demonstrate the same poor outcomes [1].

But what about oprelvekin in CIT? Unlike the other growth factors, oprelvekin was generally poorly tolerated, with numerous proinflammatory-type side effects, such as fevers and myalgias, along with a nontrivial incidence of fluid retention and atrial arrhythmias [2]. This ultimately led to its withdrawal from the market by the manufacturer in the United States. So, naturally, the development of better-tolerated thrombopoietic agents proceeded, and recombinant human thrombopoietin (rhTPO) and pegylated human megakaryocyte growth and development factor entered CIT trials. Unlike oprelvekin, these agents acted directly on c-MPL and were well-tolerated, with a minimal side-effect burden (better tolerated than even G-CSFs) [3,4]. Of course, after a handful of patients treated with pegylated human megakaryocyte growth and development factor developed antidrug neutralizing antibodies capable of cross-reacting with endogenous thrombopoietin [5], development of both of these agents was halted in the West, even though rhTPO did not cause this immunologic complication. The postscript to this story is very different depending on your country of origin. If you are in China, then, of course, rhTPO completed development for CIT, received Chinese regulatory approval, and has become integrated into Chinese oncology supportive care guidelines and routine CIT management, akin to the use of G-CSF for chemotherapy-induced neutropenia [6]. If you are outside of China, then the development of CIT treatments was completely halted for about 2 decades, during which time the use of thrombopoietic growth factors to support optimal chemotherapy treatment of patients with cancer was all but forgotten. The initial thrombopoietin receptor agonists (TPO-RAs) romiplostim and eltrombopag, which were designed explicitly to avoid the potential for cross-reacting antidrug neutralizing antibodies, were developed in and approved for immune thrombocytopenia (ITP) rather than CIT [4]. Romiplostim and eltrombopag were FDA-approved for ITP in 2008, and it took a full 14 years after this for a phase 3 trial of a TPO-RA in CIT to be published (a negative trial of avatrombopag in nadir CIT; more on this to come) [7]. Though it has been nearly 3 decades since the approval of oprelvekin, the CIT treatment space is about to receive a large influx of data and potentially the first FDA approvals in >20 years [8,9].

## 3 Why Does CIT Matter?

Hematologists are taught that, in the absence of complicating factors or tissue injury, hemostasis is generally normal in thrombocytopenic states until the platelet count drops to under 20 × 10^9^/L, or even 10 × 10^9^/L. We see this every day in increased platelet turnover states like ITP, in which bleeding is quite rare at platelet counts above 20 × 10^9^/L, unless the patient is on antithrombotic therapy or has another hemostatic stressor. Hypoproliferative thrombocytopenia, while having a higher bleeding risk than states such as ITP, is also generally safe at platelet counts above 20 × 10^9^/L, again unless the patient is on antithrombotic therapy or has another hemostatic stressor. CIT, of course, is best characterized as a hypoproliferative thrombocytopenia. For this reason, there is a common misconception that platelet counts of 10 to 20 × 10^9^/L are perfectly safe in CIT, without a significant increase in bleeding risk, and that the use of thrombopoietic support in these patients is largely unnecessary, or “treating the physician” rather than treating the patient.

Of course, this is not true, because patients with cancer are not the same as patients with ITP or aplastic anemia. Patients with solid tumors, the primary focus of CIT treatment, have aggressive, growing masses invading organs and tissues. These tumors disrupt mucosal surfaces, including the gastrointestinal and genitourinary tracts, and erode blood vessels. Patients with cancer have indwelling lines, frequent minor and not-so-minor procedures, suboptimal nutritional status, and polypharmacy. It is not surprising that the risk of clinically significant bleeding is much higher in these patients at baseline, even without antithrombotic therapy [10]. Then, when factoring in the thrombophilic state of malignancy and the frequent need for antithrombotic therapy used as primary or secondary thromboembolic prevention, the bleeding risk rises even higher. The higher risk in these patients is not purely theoretical; a study evaluating 1262 chemotherapy cycles in patients with solid tumors complicated by CIT found major bleeding in 3.4% of cycles and clear, progressive elevations in major bleeding risk when platelet counts dropped below 30 to 40 × 10^9^/L [11]. Major bleeds in sick cancer patients, when not fatal, often lead to prolonged hospital admissions, postponement of anticancer treatment, disease progression, and poor outcomes. Therefore, as would be expected, the aforementioned study found drastic reductions in overall survival in the patients with CIT who experienced major bleeds [11]. While a major bleeding rate of 3.4% may not seem so high, this is the rate *per cycle* affected by CIT, not per patient. Patients with tenuous platelet counts receiving indefinite therapy with FOLFIRI (fluorouracil, irinotecan, folinic acid) for metastatic colorectal cancer, for example, rack up cumulative bleeding risk quickly, receiving cycle after cycle, particularly given that persistent CIT often progresses with more and more chemotherapy cycles as the bone marrow progenitor pool becomes more and more depleted.

Of course, oncologists do not want their patients to bleed, and so they delay chemotherapy and dose-reduce chemotherapy—thereby reducing RDI—in an effort to prevent profound thrombocytopenia during a cycle that could lead to a bleeding event. We know very plainly that RDI reduction is detrimental to patient outcomes [[12], [13], [14], [15], [16]]. Returning to the example of FOLFIRI in colorectal cancer, a rather modest reduction in irinotecan RDI of just 20% has been demonstrated in a pooled analysis of multiple prospective clinical trials to be associated with significant reductions in both progression-free survival (hazard ratio for disease progression, 3.18; 95% CI, 1.47-6.88; *P* < .01) and overall survival (hazard ratio for death, 2.72; 95% CI, 11.22-6.04; *P* < .01) [16]. The same findings have been demonstrated in many other studies across tumor types and chemotherapy regimens [[12], [13], [14], [15], [16], [17]]. Put simply, if chemotherapy is indicated to treat a patient’s cancer, and the patient is of adequate performance status to receive it, they should receive the treatment at the studied intended doses if at all possible. This is a noncontroversial foundational principle of medical oncology.

A question that is often asked in CIT management is, “Will the use of a thrombopoietic agent increase the chance of patients living longer if higher platelet counts allow for maintained RDI and fewer deaths from bleeding?” We do not have a randomized trial that answers this question at present and likely never will, because it is infeasible to design a prospective randomized trial aimed at evaluating overall survival in CIT (it would require all patients enrolled to have one tumor type at the same stage, all receiving the same chemotherapy regimen, all have a similar performance status and be in a similar place in their cancer treatment journey, and for this standard of care chemotherapy regimen to remain unchanged over the course of the trial; this is completely infeasible from a practical trial design and enrollment standpoint), and it is unethical not to address profound thrombocytopenia in these patients with platelet transfusions when it is detected. Major randomized, placebo-controlled trials of romiplostim and avatrombopag in CIT are underway, and the results from these trials are needed to confirm the findings from early-phase and observational studies regarding the utility of TPO-RAs to maintain RDI. Real-world evidence may ultimately allow for overall survival analyses to be performed, demonstrating a survival benefit of TPO-RA management in CIT. But assuming a good safety profile of TPO-RAs in patients with cancer (discussed in detail below), we know that preserving RDI and reducing major bleeding results in better overall survival in these patients, as demonstrated by a wide body of evidence.

## 4 But what About the Potential Risks of Thrombopoietin Agonism?

Thrombopoietin agonists have potential thromboembolic risk and theoretical risks of promoting clonal hematopoietic evolution or myelofibrosis. After the erythropoiesis-stimulating agent story in cancer, the question of whether thrombopoietic growth factors could stimulate tumor growth has (appropriately) been asked. On top of this, there is now an emerging body of evidence that suggests that platelets may play a role in cancer cell metastasis. How do we reconcile these potential risks with the use of TPO-RAs for CIT?

First, we will consider thromboembolic risk, which is generally the most commonly raised concern with the use of TPO-RAs. Romiplostim, eltrombopag, and avatrombopag do not cause platelet hyperreactivity, increased platelet activation, or spontaneous platelet aggregation in patients with ITP [[18], [19], [20]]. Most studies of TPO-RAs were completed in ITP, and no randomized, controlled trial performed in this setting found meaningfully increased thromboembolic risk or was halted due to thromboembolic complications [21]. On the other hand, a randomized, controlled study of eltrombopag in patients with chronic liver disease was halted due to an excess of thromboembolic events in the eltrombopag arm, though this was not seen in numerous other randomized, controlled trials of avatrombopag or lusutrombopag in the same patient population [22]. Given the substantial baseline thromboembolic risk in patients with cancer, could there be a synergistic increase in this risk with the addition of a TPO-RA? The available randomized trials in CIT, including a 60-patient phase 2 study of romiplostim and a 122-patient phase 3 study of avatrombopag, among others, did not demonstrate an increased thromboembolic risk in patients receiving the active drug [7,23,24]. A large observational study of 173 patients with solid tumors and lymphoid malignancies receiving romiplostim for CIT, as well as other published smaller studies, observed no increased risk [[25], [26], [27]]. All of these studies observed thromboembolic rates similar to historical, comparable cancer patient populations.

Second, we will address concerns of clonal evolution and myelofibrosis. Historical concerns regarding the potential for leukemogenicity, promotion of hematopoietic clonal evolution, and development of irreversible myelofibrosis with the use of TPO-RAs have been laid to rest in ITP and myelodysplastic syndrome studies with extended-duration use of TPO-RAs and extended-duration follow-up [28,29]. In patients with known myelodysplastic syndrome, the use of TPO-RAs to treat thrombocytopenia causes a temporary increase in bone marrow blast counts, which normalizes after discontinuation of the TPO-RA. Up to 5 years of safety follow-up in randomized clinical trials have shown no higher rates of disease evolution in patients receiving TPO-RAs [28]. Similarly, no signal for any of these complications has been observed in any CIT study of TPO-RAs.

Finally, what about the potential for thrombopoietin agonism to promote tumor growth? Multiple well-designed studies evaluating thrombopoietic agents in various tumor cell lines have shown no impact of thrombopoietin agonism on tumor growth [30]. How about the concern that platelets may play a role in cancer cell metastasis? These data are intriguing and important [31], but it is preclinical and cannot drive clinical decision-making. Assuming that these data are accurate, does the specific platelet *count* matter, or is the *presence* of platelets all that matters? If the specific platelet count matters, how large a difference is needed for a higher platelet count to increase the likelihood of cancer spread relative to a lower platelet count? For example, does a platelet count of 40 × 10^9^/L reduce the risk of cancer spread relative to a platelet count of 110 × 10^9^/L? Should we add anagrelide to adjuvant and neoadjuvant chemotherapy regimens to minimize the risk of metastatic spread? (The answer to the last question, of course, is no, but it makes a point.) Thrombocytopenia has not been associated with reduced cancer metastasis or improved survival in patients with cancer, and, in fact, has been associated with a worse prognosis [32]. The goal of thrombopoietic support for patients with CIT is to maintain a platelet count around the low end of the physiologic range, rather than inducing supraphysiologic or even high-normal platelet counts.

Ultimately, then, the benefits of TPO-RA use, given the accumulated data at present, are expected to considerably outweigh the risks in patients who are expected to benefit from RDI maintenance and bleed prevention.

## 5 Who with CIT Should, and who Should not, Receive Thrombopoietic Support?

In general, patients with solid tumors who develop *persistent CIT* [33]—a platelet count of ∼50 to 100 × 10^9^/L measured on day 1 of a chemotherapy cycle—are likely to derive benefit from TPO-RA support. These patients are at high risk of immediate or eventual RDI reduction, given the persistence of their CIT past the end of the prior cycle during which it should have resolved. These patients are usually managed in 1 of 2 ways upon presentation with persistent CIT on a cycle day 1. Either they proceed with receipt of dose-reduced chemotherapy on cycle day 1 due to thrombocytopenia, or treatment will be delayed a week or more to allow additional time for platelet recovery and administration of full-dose chemotherapy. Either path results in an RDI reduction, and in the case of the delay, the persistent CIT is very likely to recur again on day 1 of the following cycle, prompting a dose reduction or another delay. Due to the progressive nature of CIT in most patients, recurrence of CIT, despite dose reductions, is common, resulting in additional dose reductions, discontinuation of one agent from a multiagent regimen (eg, going from FOLFIRINOX [fluorouracil, irinotecan, oxaliplatin, and folinic acid] to FOLFOX [fluorouracil, oxaliplatin, folinic acid] or FOLFIRI to 5-fluorouracil/leucovorin), or discontinuation of the regimen altogether. Introduction of thrombopoietic support, in the vast majority of cases, halts this vicious cycle and even reverses it [24,25], allowing for the restoration of full chemotherapy RDI—the earlier this intervention takes place, the better, particularly if a patient is being treated with a set number of cycles with curative intent. There is existing prospective phase 2 and observational evidence for the use of romiplostim in persistent CIT. Additionally, 2 pivotal phase 3 randomized, placebo-controlled trials (RECITE, Study of Romiplostim for Chemotherapy-induced Thrombocytopenia in Adult Subjects with Gastrointestinal, Pancreatic or Colorectal Cancer [NCT03362177] and PROCLAIM, Study of Romiplostim for Chemotherapy-induced Thrombocytopenia in Adult Subjects with Non-small Cell Lung Cancer, Ovarian Cancer, or Breast Cancer [NCT03937154]) are anticipated to provide conclusive evidence of the efficacy of romiplostim in maintaining RDI and further safety data in the CIT patient population. A phase 2 randomized, placebo-controlled trial of avatrombopag in persistent CIT (ACT-GI, Avatrombopag for Chemotherapy-induced Thrombocytopenia in Gastrointestinal Cancers [NCT05772546]) may demonstrate the efficacy of avatrombopag in persistent CIT, potentially offering patients an oral option. Given that no TPO-RA is currently licensed anywhere in the world for CIT (even in China, rhTPO remains the only licensed thrombopoietic agent for CIT) and use remains off-label, participation in clinical trials is more appropriate than off-label use if such a trial is available to the patient and they are eligible. In most cases, a trial is not available, and romiplostim is used off-label. Romiplostim is currently the only TPO-RA appropriate for routine off-label use because it has by far the largest body of evidence for the treatment of persistent CIT. All of these recommendations are consistent with those for the management of CIT from the National Comprehensive Cancer Network [34] and the International Society on Thrombosis and Haemostasis Scientific and Standardization Subcommittee on Hemostasis and Malignancy [35].

Patients with no history of clinically relevant CIT do not require TPO-RA support. Although there was initially interest in CIT prevention with the use of eltrombopag in patients receiving highly myelosuppressive regimens, this approach has been abandoned after multiple underwhelming clinical trials [36]. There is no reason to expose unselected patients with cancer to the risks (minimal though they may be) and the financial and patient time costs (which are not minimal) of unnecessary thrombopoietic support, and it is easy to initiate a TPO-RA once a patient develops clinically relevant CIT. The costs of TPO-RA use in CIT are highly variable, depending on the country of origin, but could result in significant financial toxicity if the agents are not covered by payers or if copays are significant.

Finally, some patients develop what is known as *nadir CIT* [33]. In contrast to the aforementioned *persistent CIT*, nadir CIT is characterized by the occurrence of a larger than expected platelet count nadir midcycle with recovery to normal or near-normal counts by the subsequent cycle day 1. Most clinically insignificant nadir CIT goes unrecognized because platelet counts are not routinely measured during the cycle count nadir. It may be recognized if a toxicity laboratory check is scheduled (to evaluate for CIT or some other reason) or if a patient presents with bleeding. Unlike persistent CIT, nadir CIT often does *not* recur. In a phase 3 randomized, placebo-controlled trial of avatrombopag in patients with nadir CIT, patients in the avatrombopag arm had improved platelet counts, but the trial was negative because a majority of the patients in the placebo arm did not experience recurrence of clinically relevant nadir CIT during the study [7]. With the knowledge gained from this trial, patients with nadir-only CIT can be considered for initiation of TPO-RA support if (1) they have particularly deep nadirs (<20 × 10^9^/L), especially if recurrent, or (2) they have a history of intracycle bleeding episodes with a documented nadir of <50 × 10^9^/L. Patients requiring antithrombotic therapy with recurrent nadir CIT may be considered for initiation of TPO-RA support at slightly higher thresholds.

This approach is summarized in the Figure. The details of agent selection, dosing, monitoring, dose modification, toxicity management, and TPO-RA discontinuation for adverse events, futility, or completion of chemotherapy are described in full detail in a recently published comprehensive review article [36], which is available to all as an open-access article on PubMed Central.

**Figure:**
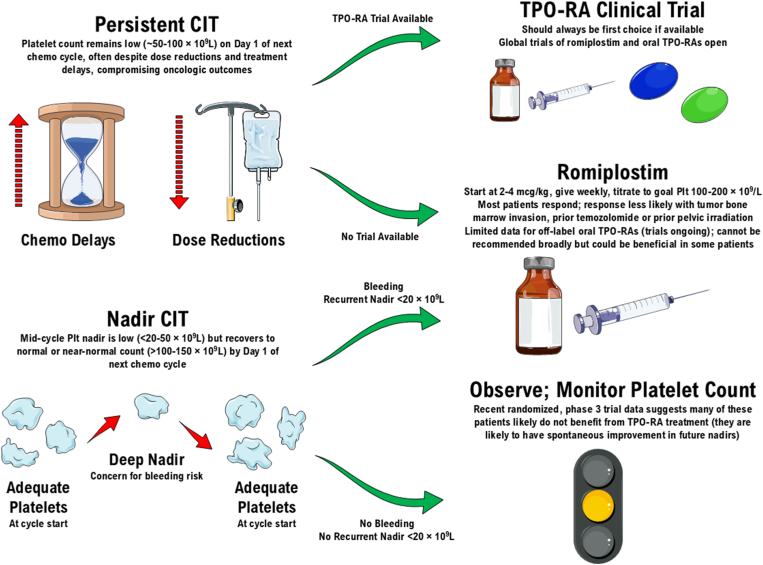
Graphical summary of nadir chemotherapy-induced thrombocytopenia (CIT) and persistent CIT, along with the author’s recommended treatment algorithm when thrombopoietin receptor agonist (TPO-RA) therapy is considered. Adapted with permission from Al-Samkari [23]. Plt, platelet.

## 6 Conclusions

Should TPO-RAs be used to treat CIT? It depends. In patients with persistent CIT, the current body of evidence, including both benefits and risks, strongly suggests that the answer is yes, to allow for RDI maintenance and bleeding prevention in this highly vulnerable patient population. In patients with nadir CIT, the answer is maybe; TPO-RAs should generally be limited to those patients with nadir CIT with recurrent profound nadirs and/or a history of bleeding associated with a substantial platelet nadir. Finally, there is no role for the use of TPO-RAs in unselected cancer patients who have not yet developed CIT in an effort to prevent it. Major randomized, placebo-controlled trials of romiplostim and avatrombopag in persistent CIT are underway and will further augment our understanding of how to best manage this common and challenging hematologic complication of malignancy.
